# Pharmacokinetic and Pharmacodynamic Modeling of Antibody-Drug Conjugates

**DOI:** 10.3390/cancers18010005

**Published:** 2025-12-19

**Authors:** Patrick M. Glassman

**Affiliations:** Department of Pharmaceutical Sciences, Temple University School of Pharmacy, Philadelphia, PA 19140, USA; patrick.glassman@temple.edu; Tel.: +1-215-707-0355

**Keywords:** antibody-drug conjugates, pharmacokinetics, pharmacodynamics, PK/PD modeling

## Abstract

Antibody-drug conjugates (ADCs) have risen in prominence over the past 15 years, with numerous regulatory approvals in oncology. A complicating factor in the development of ADCs is the presence of numerous analytes with unique pharmacologic properties. Following administration, ADCs are present in the body as the intact ADC, unconjugated antibody, and liberated payload. Due to heterogeneity in conjugation and in vivo deconjugation rates, the drug-to-antibody ratio (DAR) changes with time. Each of these molecular species has unique pharmacokinetic (PK) and pharmacodynamic (PD) properties that should be understood and characterized. One approach that is frequently applied is the development of in silico mathematical models to characterize and predict the PK/PD of ADCs. In this review, we summarize key mechanisms controlling the PK/PD of ADCs. This provides context for a detailed discussion of the array of PK/PD models that have been applied for ADCs, ranging from empirical compartmental models all the way through system-level models, such as physiologically based pharmacokinetics (PBPK) and cell-level PK/PD models. We provide a critical discussion of the strengths, weaknesses, and utility of each of these model structures.

## Simple Summary

Antibody-drug conjugates are a growing class of drugs that combine the selectivity of monoclonal antibodies with the potency of chemotherapeutics. They are designed to target potent drugs to tumor cells and, ideally, spare healthy cells from exposure to, and damage by, chemotherapeutics. To facilitate their development, mathematical models have been proposed that account for their pharmacokinetics and pharmacodynamics, with a goal of predicting clinical behavior and optimizing doses. In this review, a summary of mechanisms controlling the behavior of antibody-drug conjugates in the body is provided, followed by a detailed discussion of the different classes of models that have been applied in the literature for antibody-drug conjugates.

## 1. Introduction

The use of antibodies as carriers for cytotoxic payloads, whether they be chemotherapeutic agents [1,2] or radioisotopes [3], has been described for over 50 years, with the first studies being published before the seminal Köhler and Milstein article describing monoclonal antibody (mAb) technology [4]. These early efforts evolved into the rapidly expanding class of drugs known as antibody-drug conjugates (ADC). Critical advances enabling the development of safe and efficacious ADCs include hybridoma technology, antibody humanization, and, perhaps most importantly, the development of linker technology that minimizes pre-emptive release of cytotoxic agents [5]. Currently, there are 13 FDA-approved ADCs used to treat a diverse array of cancers, as well as 1 FDA-approved immunotoxin, with many additional ADCs in clinical development [6].

**Figure 1:**
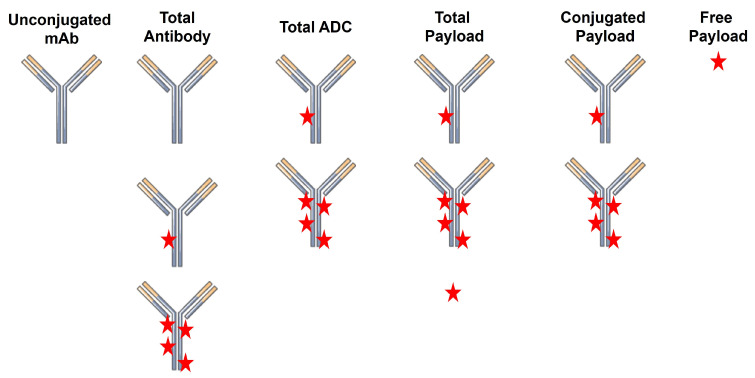
ADC-related analytes that can be quantified during pharmacokinetic studies.

A critical challenge in the successful development of ADCs as therapeutics is the characterization of their pharmacokinetics (PK) and pharmacodynamics (PD). This is in no small part due to the high complexity of the final ADC product. Unlike small molecule drugs and unconjugated biologics, ADCs have a high degree of molecular heterogeneity. ADCs consist of an array of molecules with a range of drug-to-antibody ratios (DAR), typically following a normal distribution centered on a DAR of ~4. Following injection, there are several analytes that can be quantified, including total antibody (TAb; unconjugated + conjugated), total ADC (tADC), total drug, free drug, and unconjugated antibody (Figure 1). Quantification of the concentrations of individual DAR species can also be performed to assess in vivo deconjugation.

In this review, the mechanisms underlying the PK/PD properties of ADCs will be briefly reviewed to provide critical context. This is followed by an extensive discussion of empirical and mechanism-based PK/PD models that have been developed for ADCs, highlighting their features, strengths, weaknesses, and applications.

## 2. ADC Pharmacokinetics

### 2.1. Absorption

All FDA-approved ADCs are dosed via intravenous infusion; however, there is burgeoning interest in the use of extravascular routes of administration, namely subcutaneous (SC) injections. A recent report examined the PK of monomethyl auristatin E (MMAE)-conjugated trastuzumab in tumor-bearing mice. A critical finding was local toxicity at the SC injection site; nonetheless, ADCs were absorbed slowly with a t_max_ of 24 h and a reduction in C_max_ of >70% relative to intravenous dosing. There was reasonable bioavailability of the ADC (≥50% in mice) [7]. The relative paucity of studies investigating the SC absorption of ADC makes it challenging to attribute a given mechanism to their absorption; however, it is likely that ADC absorption follows similar mechanisms as unconjugated mAbs, with absorption being primarily via the lymphatics [8,9].

### 2.2. Distribution

The primary physiologic drivers of tissue distribution of ADCs are similar to mAbs, as their molecular size precludes diffusion across the endothelial barrier. Therefore, tissue entry of ADCs is typically assumed to be via convective uptake, driven by transendothelial fluid flow and the relative size of the ADC to paracellular pores. On a molecular basis, a key driver of tissue distribution of ADC is the DAR, as experimental data have shown a positive correlation between DAR and volume of distribution (V_ss_) in mice, with V_ss_ increasing ~5-fold between a DAR2 ADC and a DAR10 ADC for maytansinoid ADC [10]. This was attributed to hepatic uptake, as high DAR ADC had 2–4-fold higher concentrations in the liver within 2 h of injection [10]. Molecular charge has also been shown to affect tissue uptake, with MMAE-conjugated trastuzumab having enhanced tissue uptake when trastuzumab was engineered to have a positive charge, as shown by a near doubling of V_ss_ and a 1.5–9-fold increase in the tissue-to-plasma AUC ratios for several organs (liver, spleen, and tumor xenograft) in mice [11].

### 2.3. Non-Specific Elimination

Elimination of the mAb component of an ADC is expected to be driven by similar factors as an unconjugated mAb, with non-target-dependent elimination occurring intracellularly following fluid-phase uptake (pinocytosis). The efficiency of this elimination pathway is muted by interactions with the neonatal Fc receptor (FcRn) [12]. In vitro studies suggest that increases in DAR lead to improvements in the equilibrium FcRn binding affinity for MMAE-based ADC; however, these results have not been followed up with in vivo studies to confirm if there are any changes in PK [13]. Following intracellular elimination of the mAb, the liberated payload may be effluxed or diffuse out of the cell and circulate with its own unique PK properties and mechanisms, which would be more typical of a small molecule drug. This may lead to the need for studies of metabolic or transporter-mediated drug–drug interactions based on exposure of the liberated payload and relevant FDA guidances.

### 2.4. Target-Mediated Elimination

**Figure 2:**
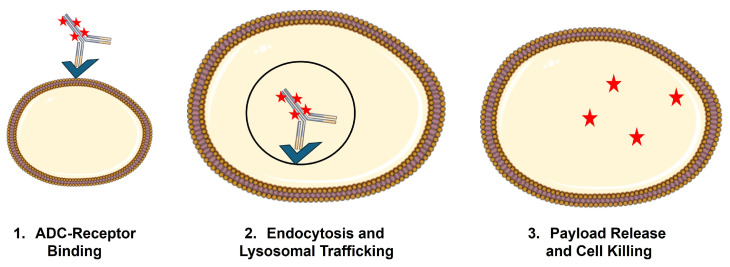
Schematic of receptor-mediated uptake of ADC by target-expressing cells.

For ADCs, efficacy and elimination are often impossible to deconvolute, as binding and internalization of ADCs by the target cell generally result in lysosomal catabolism of the ADC and liberation of the cytotoxic payload (Figure 2). This phenomenon, referred to as target-mediated drug disposition (TMDD), was first proposed by Gerhard Levy in 1994 [14] and mathematically formalized by Mager and Jusko in 2001 [15]. While a detailed discussion of the general PK expectations for molecules that are subject to TMDD is beyond the scope of this review, it is anticipated that TMDD will lead to dose-dependent changes (usually decreases) in the primary PK parameters, clearance (CL), and volume of distribution (V). This is due to a significant fraction of the administered dose being eliminated via interaction with the pharmacologic target. In addition, if treatment results in target depletion, as would be the goal when using ADCs, there will be non-stationary PK, with the impact of target binding decreasing upon multiple dosing, resulting in increased exposure on later doses [16]. TMDD is a critical PK/PD driver for ADC, as uptake by target-expressing cells and payload liberation inside these cells is a necessary condition for ADC to achieve their selective cytotoxicity on tumor cells.

### 2.5. Deconjugation

An additional mechanism by which ADCs are eliminated from the body is the deconjugation of the payload from the mAb. While the design of a linker with minimal systemic cleavage is a critical feature enabling the successful use of ADC, some payload release in the circulation is unavoidable. This has been incorporated into PK/PD models of ADC (Section 3) to account for the changes in DAR in the circulation. If all of the drug molecules are deconjugated from a given molecule of ADC prior to elimination of the antibody itself, it is anticipated that the deconjugated mAb will have PK features similar to the parental, unconjugated mAb.

## 3. Pharmacokinetic/Pharmacodynamic Modeling of ADC

**Figure 3:**
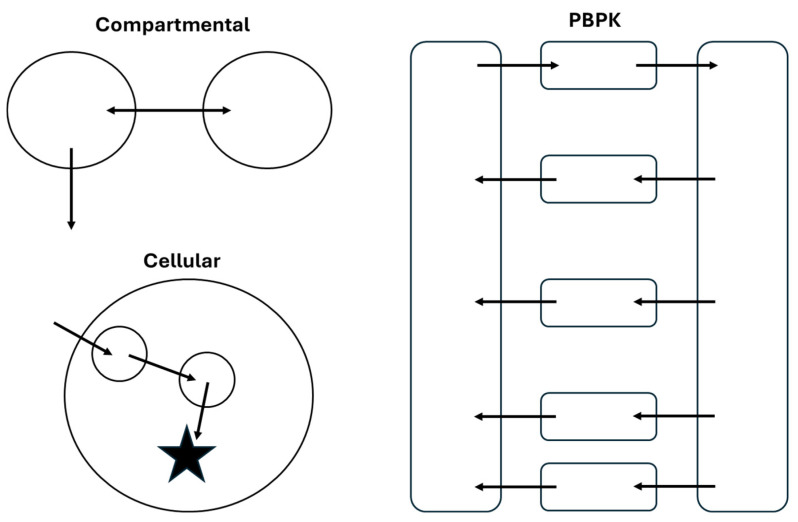
Comparison of structural features of compartmental, PBPK, and cellular models of ADC PK/PD. These structures are representative of model structures that could be used for any component of an ADC that is quantified during a PK study.

Characterization and prediction of the PK/PD profiles of ADCs has been achieved using several model structures, ranging from empirical compartmental models to physiologically based pharmacokinetic (PBPK) models. A high-level comparison of the structure of these models is shown in Figure 3. As with any modeling effort, selection of the model structure and complexity should be based on the overall goals of the project. In this section, frequently used model structures for ADCs will be discussed with examples from the literature to highlight the utility of different approaches.

**Figure 4:**
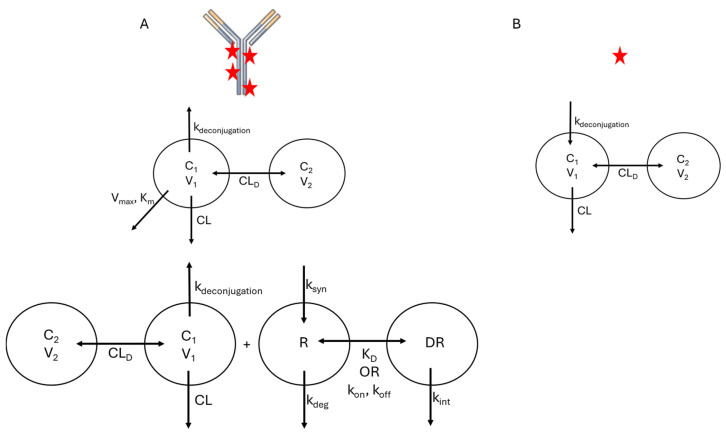
Typical Compartmental Model Structures for ADC. (**A**) Frequently used model structures for total ADC PK. Upper: Compartmental model with parallel linear (CL) and Michaelis–Menten (V_max_, K_m_) elimination. Lower: Full TMDD model. (**B**) Frequently used model structure for the released payload. Payload kinetics are driven by release from the ADC (k_deconjugation_). For both modeled species, the number of distribution compartments (e.g., 1-compartment vs. 2-compartment) is governed by the observed data.

While the model structures that are typically used for ADC will resemble those used for other biologics and small-molecule drugs, there are unique features of the ADC that complicate model development. As discussed in Section 2, ADCs are a dynamic, heterogeneous mixture of molecules, and specific bioanalytical assays are required to quantify distinct molecular species in vivo. Based on what analytes are quantified during PK studies, PK/PD models for ADCs often include compartments and specific parameters representing TAb, tADC, total drug, free drug, and unconjugated antibody. This is necessary because each of these species may have distinct PK features due to differences in molecular properties, analogous to a PK model that accounts for a small molecule drug and its metabolite(s). Schematically, these would appear as overlaid models with individual structures that resemble those shown in Figure 3 and Figure 4 but connected by rate constants that describe drug deconjugation. In many cases, individual DAR species are not modeled separately, as these are not often quantified in vivo; however, several examples are described below where the transition between DAR species was monitored and modeled. In the sections below, the literature examples are highlighted that account for different analytes, with descriptions of how their differential PK behavior is accounted for in the models.

### 3.1. Compartmental Models

#### 3.1.1. Compartmental Model Structures

As the disposition mechanisms are largely conserved between mAbs and ADCs, structural features of compartmental models are typically similar between these drug classes. Typically, a 2-compartment mammillary model is sufficient to describe the disposition of ADC, with elimination being described using either linear or non-linear (Michaelis–Menten, TMDD) kinetics, or a combination of the two (Figure 4). However, a key feature that is often integrated into compartmental models for ADC is a term related to deconjugation kinetics, which will be the focus of this section. Deconjugation and liberation of free payload is often described in two manners. One approach is to discretely model each DAR species present in vivo with deconjugation reactions, releasing free drug molecules. As individual DAR species are not typically measured in PK studies, a single deconjugation rate and set of disposition parameters are used, and the sum total of the different DAR species represents the total ADC in vivo. An example of this approach was used to describe the PK of trastuzumab emtansine (T-DM1) in both cynomolgus monkeys [17] and humans [18]. In models that follow this structure, the distribution of DAR species measured prior to administration is used to provide initial conditions for each species in vivo. One advantage of using this model structure is that it can be used to provide inference into changes in both the average and distribution of DAR in vivo. However, as different DAR species are not usually measured in plasma, validation of the assumptions of this model is challenging. One example where bioanalytical assays enabled model validation measured the in vitro deconjugation rates of T-DM1 and individual DAR species in rat and cynomolgus monkey plasma to identify rate constants for each deconjugation step and then used those rates as parameter values in a PK model [19]. This was expanded in a model that accounted not only for individual deconjugation rates between DAR species, but also modeled changes in clearance as a function of DAR, with total CL increasing as a function of DAR using an exponential function [20]. This was made possible due to the measurement of individual DAR species in plasma samples, providing PK profiles for DAR0, DAR2, DAR4, and DAR6 ADC for an anti-HER2-MMAF ADC. A more frequently used approach, particularly in clinical trials, is to use a single deconjugation rate that represents a fraction of the total CL of the ADC, which produces free payload. This simpler model structure is generally able to characterize the PK of total ADC and free payload [21,22].

#### 3.1.2. Population Models

Population PK (PopPK) models are routinely used during the clinical phase of development for ADCs (and for almost every therapeutic). These models typically utilize one of the compartmental model structures discussed in Section 3.1.1 as a base. However, the unique feature of PopPK models is that they use patient-specific features (covariates) to describe inter-individual differences in PK. A summary of published PopPK models for ADCs is shown in Table 1. While an exhaustive description of the results of each of the studies is beyond the scope of this review, several key observations can be made. First, the majority of models consider, at a minimum, the PK profiles of tADC and the liberated payload, as these are key drivers of therapeutic response and toxicities in the clinic. Second, the typical structural model for an ADC is similar to that which is usually utilized for mAbs, a 2-compartment model with elimination mechanism dictated by the observed data. In the studies summarized in Table 1, any profiles that had non-linearities were described using Michaelis–Menten elimination in lieu of a TMDD model. This is likely due to the ease of model fitting and adequate model performance with this simplified model structure. Third, while many covariates are reported, several appear frequently in analyses, which are consistent with reports of PopPK modeling of mAbs [23,24]. In short, the most frequently appearing covariates include metrics of body size (weight, body surface area, lean body weight, ideal body weight), demographic characteristics (biological sex, race/ethnicity), and readily measured biomarkers (serum albumin concentrations, tumor burden). In models that consider PK of the liberated payload, metrics of liver (bilirubin, AST, hepatic impairment score) and kidney (creatinine clearance) function appear more frequently as covariates, as these are reflective of the functional status of eliminating organs for small molecule drugs. In general, PopPK models for ADC are useful in characterizing the behavior of ADC in a diverse patient population, allowing identification and quantification of patient-specific factors that may require dose adjustments to achieve optimal patient outcomes. However, caution must be taken with these models if investigators wish to extrapolate beyond the range of ADC doses/plasma concentrations used for validation.

### 3.2. Physiologically Based Models

PBPK models integrate determinants of drug disposition across scales and can be used to predict the plasma and tissue PK profiles of a range of molecules. In general, a PBPK model for ADC would include, at a minimum, two distinct, linked models, one for the ADC and the other for liberated payload, which are linked via a first-order deconjugation rate constant. No examples have been reported that developed PBPK models for individual DAR species. A list of PBPK models discussed in this section can be found in Table 2. Cilliers and colleagues used a Krogh cylinder to model the intratumoral distribution of tADC [46]. This feature allowed for the prediction of the depth of penetration of trastuzumab emtansine (T-DM1) in the presence and absence of unconjugated trastuzumab. Model predictions of increased penetration depth when the two molecules were co-administered were verified experimentally using microscopy, demonstrating the utility of this approach in overcoming the binding site barrier [46]. Khot et al. also developed a PBPK model for T-DM1 in rats, which explicitly accounted for the deconjugation kinetics of the ADC in different physiological spaces, as well as the binding of the drug to its intracellular target. This model was able to accurately predict plasma and tissue concentrations of T-DM1 in rats, and scaling of the model to humans allowed for reasonable predictions (% prediction error < 50%) of plasma PK of T-DM1, free DM1, and total trastuzumab [47]. The same group has developed individual models for MMAE [48] and trastuzumab-MMAE (T-vc-MMAE) [49], which were able to accurately characterize the PK of T-vc-MMAE (free and conjugated) and total trastuzumab in tumor-bearing mice. This model has recently been scaled to rats, monkeys, and humans and used to predict the PK of a range of MMAE-containing ADC across species, with accurate characterization of changes in DAR with time in all three species [50]. More recently, a PBPK/PD model has been developed for trastuzumab deruxtecan (T-Dxd) and sacituzumab govitecan that accounts for topoisomerase inhibition and tumor growth kinetics. This model was able to accurately predict PK and tumor growth inhibition in mice, as well as plasma PK, progression-free survival, and risk of interstitial lung disease as an adverse effect in humans [51]. Taken together, this shows that PBPK has strong predictive capacities for ADC and is able to integrate mechanisms controlling the PK of both the intact ADC and liberated payload. This presents unique advantages over other model types as it allows more ready projection of the clinical PK of ADC, as well as allows the use of a single ADC model structure to predict the PK of ADC with distinct payloads by simply swapping out the model for free drug, as in [47,49,50]. However, the development and validation of PBPK models is much more technically challenging than more empirical model structures, so their general use is limited at the current time.

#### Minimal Physiologically Based Models

Minimal PBPK (mPBPK) models have been implemented for mAbs to describe their plasma PK across species [52,53,54,55,56,57]. Briefly, mPBPK models for biologics lump organs together based on the status of the vasculature, namely whether it is ‘tight’ or ‘leaky’, using the sum of organ volumes and perfusions to drive distribution into organs. However, unlike PBPK models described above, mPBPK models are generally used to describe PK in plasma or serum and not in tissues. However, several extensions have been built to the mPBPK model to describe mAb PK in the target tissue, including examples for the kidney [58], synovial fluid [59], skin [60], and the brain [61]. A mPBPK model was proposed for T-DM1 that extended the base mPBPK structure to include a tumor, which was described using a Krogh cylinder to account for tumor penetration of ADC. This model was able to capture the plasma PK of T-DM1 and was used to make predictions regarding the impact of dosing strategies, payload potency, and target expression on tumor penetration and tumor cell killing [62].

### 3.3. Cell-Based Models

As cell binding, internalization, and trafficking are critical to the PK/PD of ADCs, there have been efforts made to develop cell-level PK/PD models. These models can then be integrated into any of the model structures described above to provide more mechanistic insights into the behavior of the ADC at the target site, typically a tumor. Basic cell processing models for ADCs include parameters such as receptor binding kinetics, internalization, degradation, and efflux of liberated payload. This would allow for the identification of engineerable parameters that could improve the exposure of tumor cells to the free payload. In one published example, a sensitivity analysis showed that internalization rate and drug efflux rate were the most critical parameters controlling delivery of payload to cells in vitro [63]. This model structure has been integrated into PK/PD models for ADCs, with tumor cell binding and internalization, tubulin binding, and cell growth and killing being included in the tumor model sub-structure [64,65]. These tumor models are either integrated into a compartmental model as a separate tumor compartment or replace the tumor compartment in a PBPK model. Additionally, they can be used as standalone models to characterize the behavior of ADCs in cell culture systems. A series of cell-based models was developed for trastuzumab-MMAE ADCs, starting with initial models of ADC and MMAE cellular PK/PD in high target-expressing cells [66] and extensions to include bystander effects in co-cultured cells with low target expression [67]. Additionally, this modeling framework was used to derive a quantitative relationship between target expression and intracellular exposure of ADCs in tumor cells, which could be used to better understand differences in PK/PD of ADCs [68]. These cell-based models were integrated into PK/PD models to predict efficacy in mouse xenograft models [69] and to project the in vivo bystander effect in tumors with heterogeneous target expression [70]. Cell-level PK/PD models can be easily included in in vivo PK/PD models, with parameters being extrapolated from in vitro to in vivo to project intratumoral PK/PD in animal models or in patients. The ability of this strategy to accurately predict the in vivo PK/PD relationship for ADC lends confidence to the relevance of the in vitro system and in silico model to the animal models being utilized. At the most complex level, systems pharmacology models have been developed to link ADC PK/PD. In one example, in vitro cell interactions (binding, internalization, degradation, and cell killing) for healthy and tumor cells were used to build a model to project ADC efficacy and dosing in mice and in humans. In this case, the tumor model accounted for the spatial distribution of ADC, using a Krogh cylinder, and linked tumor payload concentrations to a tumor growth inhibition and cell kill model to predict progression-free survival with two trastuzumab-based ADC [71]. This has been recently extended to account for intratumoral ADC kinetics with dynamic tumor growth and shrinkage [72], which would be more reflective of the in vivo scenario where treatment with ADC and tumor growth/death inextricably affect each other. Cell-based models additionally allow for the description of intracellular signaling pathways affecting ADC PK/PD and can be extended towards systems pharmacology models. The effects of ADC therapy have also been tested in quantitative systems pharmacology (QSP) models of specific tumor types to inform optimal design of combination therapy regimens [73,74].

## 4. Conclusions and Prospectus

Development of in silico models to characterize and predict the PK/PD behavior of ADC is a critical step in their overall development paradigm. The array of models that are frequently used for ADC was described, ranging from empirical compartmental models to highly mechanistic cell and physiologically based models. Selection of a model structure should, according to best practices, be determined by both the goals of the development program and the array of data that is available. Mechanistic models that probe cell trafficking and signaling following ADC treatment may have greater utility at the early discovery and preclinical development stage, where groups are interested in engineering the ADC to have optimal properties on the cell level. These engineerable properties include, but are not limited to, (1) selection of target receptor, (2) receptor interactions (binding kinetics/affinity, internalization), (3) linker design to achieve optimal payload release, and (4) selection of a payload that is expected to have high potency against the tumor type of interest. Notably, integration of these cell-based models into PBPK or compartmental models has been used to describe the intratumoral distribution of ADC in preclinical species, namely xenograft mouse models. As a molecule progresses from preclinical species into the clinic, there is often a shift from mechanistic models aimed at understanding disposition mechanisms and predicting PK in higher species towards empirical models that characterize plasma PK. This is in no small part due to the shift in availability of samples, as in preclinical species, quantification of ADC tissue distribution is feasible; however, plasma samples are typically all that is available in the clinic. These empirical models focus on quantifying the impact of patient-specific covariates on disposition, as this will inform groups if there is a need for dose adjustments in certain patients, as well as developing links between exposure and response and/or toxicity.

One area where mathematical modeling could help provide improvements in the development of ADC is the projection of clinical behavior from preclinical species. It is well-established that preclinical tumor models, particularly xenograft models, do not routinely predict clinical outcomes. This can be highlighted in one of the first prominent failures in the ADC development space, that of BR96-Dox, a conjugate between a Lewis Y-targeting mAb and doxorubicin. Early reports of this molecule touted its ability to ‘cure xenografted human carcinomas’ [75]; however, clinical development of BR96-Dox was impeded by on-target toxicities associated with Lewis Y expression in the gastric mucosa [76]. In rodent models, the target is typically only expressed in the xenografted tumor due to the lack of cross-reactivity of mAbs between human and rodent molecules. However, as the amount of available data regarding endogenous protein distribution in humans has grown through sources such as the Human Protein Atlas [77] and PaxDb [78], there is an increased ability to leverage this data to make a priori predictions of distribution in humans using PBPK modeling [79,80,81]. These approaches make it conceivable that simulations could be performed to project likely sites of tissue uptake of ADC in humans, which could then be used to inform in vitro studies to evaluate potential toxicities, as well as potential strategies to bypass on-target toxicities in healthy tissues, such as local administration of ADC or co-dosing of unconjugated mAb to block uptake in healthy organs [82].

An additional approach that could be used to project the intratumoral PK/PD of ADC would be to use sophisticated in vitro systems, such as tumor spheroids, tumor organoids, or organ-on-a-chip technology. This would have the added benefit of serving as a New Approach Methodology, which the FDA is actively encouraging for Investigational New Drug applications. While there have been a few recent studies using tumor spheroids or organoids to evaluate ADC efficacy in vitro [72,73,74], these have not yet been used to inform the development of PK/PD models. Data that would need to be acquired in these models to support PK/PD modeling would include the rate and extent of penetration, association with cells, and cell killing. Further validation of the performance of these advanced in vitro tumor models would be necessary to provide confidence in their predictive capacity for PK/PD.

In summary, the array of available PK/PD models for ADC has grown substantially over the past 15 years, largely in parallel with increased clinical use of ADCs as a therapeutic agent. These models can be deployed in a fit-for-purpose manner, allowing investigators to select model structures that are able to answer questions related to development without being overly complex and requiring excessive data and computational power to be used. As the field continues to grow, it is likely that, similar to other drug classes, PK/PD modeling will be increasingly utilized to support development teams and guide the design of studies.

## Figures and Tables

**Table 1:** Published Population Pharmacokinetic Models for Antibody-Drug Conjugates.

ADC	Disease	Model Structure	Covariates	References
Trastuzumab Emtansine	Breast Cancer	2-CM, Linear	CL: Weight, Albumin, Tumor Burden, AST V_c_: Weight	[25]
Trastuzumab Emtansine	Breast Cancer	2-CM, Linear	CL: Weight, Albumin, Shed HER2 ECD, Tumor Size, AST, Baseline Trastuzumab V_c_: Weight	[26]
Brentuximab Vedotin	Hematologic Malignancies	ADC: 3-CM, Linear MMAE: 2-CM, Linear	ADC CL, V_1_: Weight, Sex ADC V_2_, V_3_, Q_2_, Q_3_: Weight MMAE CL, Q, V_1_, V_2_: Weight	[27]
Trastuzumab Emtansine	Gastric Cancer	2-CM, Linear + M-M	CL: Weight, Baseline Trastuzumab V_c_: Weight Weight (CL, V_c_)	[28]
Brentuximab Vedotin	Lymphomas	ADC: 3-CM, Linear MMAE: 2-CM, Linear	ADC CL: BSA, Albumin, ADA Status, Tumor Type ADC V_c_: BSA MMAE CL: BSA, Albumin, Bilirubin, Creatinine MMAE V_c_: BSA	[29]
Gemtuzumab Ozogamicin	Pediatric AML	Total Ab: 2-CM, Time-Dependent CL Calicheamicin: 2-CM, Linear	Total Ab CL, V_1_, k_des_: Weight Calicheamicin CL, V_1_: Weight	[30]
Gemtuzumab Ozogamicin	AML	Total Ab: 2-CM, Time-Dependent CL Calicheamicin: 2-CM, Linear	Total Ab CL: Weight, Dose, Albumin Total Ab V_1_: Weight, Dose, Albumin, Sex Total Ab k_des_: Bone Marrow Blast, Peripheral Blast, Concomitant Medications Calicheamicin CL/F, V_1_/F: Weight	[31]
Depatuxizumab Mafodotin	Solid Tumors	Total Ab: 2-CM, Linear MMAF: 1-CM, Linear	Total Ab CL, V_c_: Tumor Type, Weight, Sex Deconjugation Rate: Weight MMAF CL: Tumor Type, Weight, Albumin, Manufacturing Process, Bilirubin, Sex	[32]
Brentuximab Vedotin	Hodgkin’s Lymphoma	ADC: 2-CM, Linear MMAE: 2-CM, Linear	ADC CL: Albumin, BSA ADC V_c_: Albumin, Sex ADC V_p_: BSA MMAE CL: BSA, CrCL, Albumin	[33]
Polatuzumab Vedotin	Non-Hodgkin Lymphoma	ADC: 2-CM, Linear + TDCL + M-M MMAE: 2-CM, Linear + M-M	ADC CL_INF_: Weight, Sex, Albumin, Combination Therapy, B-Cell Count, Tumor Size ADC V_1_: Weight, Sex, Race, Treatment Status ADC V_2_, Q: Weight ADC CL_T_: Treatment Status, Tumor Size, B-Cell Count ADC k_des_: Combination Therapy ADC Conversion Factor: Weight, Sex, Treatment Status, Combination Therapy, Hepatic Impairment, ECOG, Albumin	[34]
Trastuzumab Deruxtecan	Solid Tumors	ADC: 2-CM, Linear Deruxtecan: 1-CM, Linear	ADC CL: Weight, Albumin, Tumor Size, Race, Sex ADC V_1_: Weight, Sex ADC V_2_: Race ADC k_rel_: Treatment Cycle Deruxtecan CL: Weight, Bilirubin, AST, Concomitant Medications Deruxtecan V: BSA, Age, Drug Product	[35]
Belantamab Mafodotin	Multiple Myeloma	ADC: 2-CM, Linear Total mAb: 2-CM, Linear MMAF: 2-CM, TDCL	ADC V_1_: Weight, Albumin, Sex, Clinical Trial ADC CL: Weight, Albumin, Soluble BCMA, IgG, Dose, Clinical Trial ADC V_2_: Dose ADC k_dec_: Dose Total mAb Correction Factor: Dose, Clinical Trial MMAF V_1_: Soluble BCMA, IgG, Clinical Trial	[22]
Tisotumab Vedotin	Solid Tumors	ADC: 2-CM, Linear + M-M MMAE: 1-CM, Linear	ADC CL, V_C_: Weight, Sex, Albumin ADC Q, V_p_: Weight MMAE CL: Weight, Albumin, Tumor Type, ECOG, Hepatic Impairment MMAE V: Weight, ECOG, Albumin	[36]
Patritumab Deruxtecan	Solid Tumors	ADC: 2-CM, Linear + M-M Deruxtecan: 1-CM, Linear	ADC CL_lin_: Weight, Albumin, Sex, Tumor Type ADC V_C_: Weight ADC k_rel_: Weight Deruxtecan CL: Hepatic Impairment	[37]
Camidanlumab Tesirine	Lymphoma	2-CM, Linear + M-M	CL: Soluble CD25, Sex, Weight V_1_: Sex, Weight Q, V_2_: Weight V_max_: Soluble CD25 Deconjugation CL: Race	[38]
Mirvetuximab Soravtansine	Ovarian Cancer	ADC: 2-CM, Linear + M-M DM4: 2-CM, Linear SmDM4: 1-CM, Linear	ADC CL: AlBW, Albumin ADC V_1_, V_2_: Age, AlbW DM4 CL: AlBW DM4 k_met_: AST	[39]
Sacituzumab Govitecan	Solid Tumors	ADC: 2-CM, Linear SN-38: 2-CM, Linear Total Ab, 2-CM, Linear	ADC CL: Weight, Albumin ADC Q, V_1_, V_2_: Weight SN-38 CL/F, Q/F: Weight Total Ab CL: Weight, Albumin, Cancer Type Total Ab V_1_: Weight, Sex Total Ab Q, V_2_: Weight	[40]
Inotuzumab Ozogamicin	Pediatric ALL	2-CM, Linear, TDCL	CL: Tumor Type, Lean Body Mass, Concomitant Rituximab V_1_: Lean Body Mass TDCL_0_: Tumor Type Lean Body Mass k_des_: Tumor Type, Peripheral Blast Count, Age	[41]
Enfortumab Vedotin	Urothelial Carcinoma	ADC: 3-CM, Linear MMAE: 2-CM, Linear	ADC CL: Weight, Age, Albumin, Manufacturing Process, Sex, Tumor Burden ADC V_1_: Weight, Hemoglobin, Sex ADC Q_2_, Q_3_, V_2_: Weight ADC V_3_: Weight Analytic Laboratory, Sex MMAE CL: Weight, Albumin, ECOG, Hemoglobin, Manufacturing Process, Bilirubin ADC V_1_: Weight, Albumin, Manufacturing Process, Tumor Burden ADC V_2_: Weight, Albumin, ECOG, Hemoglobin, Manufacturing Process, Sex ADC Q: Weight	[42]
Zilovertamab Vedotin	Hematologic Malignancies	ADC: 2-CM, Linear MMAE: 2-CM, Linear	ADC CL, V_C_: Weight MMAE CL, V_c_: Weight	[43]
Farletuzumab Ecteribulin	Solid Tumors	2-CM, Linear	CL, V_1_, V_2_: BSA	[44]
Patrimumab Deruxtecan	Solid Tumors	ADC: 2-CM, Linear + M-M + TDCL Deruxtecan: 1-CM, Linear + M-M	ADC CL: Weight, Hepatic Impairment ADC CL_inf_: Weight, Country of Origin, Tumor Type, Prior Chemotherapy, Hepatic Impairment ADC V_1_: Weight, Country of Origin ADC V_2_: Weight Deruxtecan CL: Weight, Hepatic Impairment, Country of Origin, Tumor Type, Prior Chemotherapy Deruxtecan V: Weight, Country of Origin	[21]
Datopotamab Deruxtecan	Solid Tumors	ADC: 2-CM, Linear + M-M Deruxtecan: 1-CM, Linear	ADC CL_lin_: Weight, Albumin, Age, Sex, Country ADC V_C_: Weight, Sex ADC V_p_: Weight ADC V_max_: Tumor size DAR: Treatment Cycle Deruxtecan CL: Weight, Albumin, AST, Bilirubin, Country Deruxtecan V: Weight, Sex	[45]

**Table 2:** Published PBPK Models for Antibody-Drug Conjugates.

Target	Antibody	Drug	Species	References
HER2	Trastuzumab	DM1	Mouse	[46]
HER2	Trastuzumab	DM1	Rat Human	[47]
HER2	Trastzuzumab	MMAE	Mouse	[49]
Various	Various	MMAE	Rat Monkey Human	[50]
HER2 TROP-2	Trastuzumab Sacituzumab	Deruxtecan SN-38	Mouse Human	[51]

## Data Availability

No new data were created or analyzed in this study. Data sharing is not applicable to this article.

## Abbreviations

The following abbreviations are used in this manuscript:

ADA	Anti-Drug Antibody
ADC	Antibody-Drug Conjugate
AST	Aspartate Aminotransferase
BCMA	B-Cell Maturation Antigen
BSA	Body Surface Area
CM	Compartment Model
CL	Clearance
CrCL	Creatinine Clearance
DAR	Drug-to-Antibody Ratio
ECD	Extracellular Domain
ECOG	Eastern Cooperative Oncology Group
F	Bioavailability
FcRn	Neonatal Fc Receptor
FDA	Food and Drug Administration
mAb	Monoclonal Antibody
MMAE	Monomethyl Auristatin E
MMAF	Monomethyl Auristatin F
PBPK	Physiologically Based Pharmacokinetics
PD	Pharmacodynamics
PK	Pharmacokinetics
PopPK	Population Pharmacokinetics
SC	Subcutaneous
TAb	Total Antibody
tADC	Total Antibody-Drug Conjugate
TDCL	Time-Dependent Clearance
T-DM1	Trastuzumab Emtansine
T-Dxd	Trastuzumab Deruxtecan
TMDD	Target-Mediated Drug Disposition
T-vc-MMAE	Trastuzumab-MMAE
Vss	Volume of Distribution
